# Identification of biomarkers for the antiangiogenic and antitumour activity of the superoxide dismutase 1 (SOD1) inhibitor tetrathiomolybdate (ATN-224)

**DOI:** 10.1038/sj.bjc.6604226

**Published:** 2008-02-05

**Authors:** F Doñate, J C Juarez, M E Burnett, M M Manuia, X Guan, D E Shaw, E L P Smith, C Timucin, M J Braunstein, O A Batuman, A P Mazar

**Affiliations:** 1 Attenuon, LLC, San Diego, CA 92121, USA; 2 DE Shaw Research, LLC, New York, NY 10036, USA; 3 Division of Hematology and Oncology, State University of New York, Downstate Medical Center, Brooklyn, NY 11203, USA

**Keywords:** biomarkers, angiogenesis, superoxide dismutase, tetrathiomolybdate, circulating progenitor cells

## Abstract

Tetrathiomolybdate (choline salt; ATN-224), a specific, high-affinity copper binder, is currently being evaluated in several phase II cancer trials. ATN-224 inhibits CuZn superoxide dismutase 1 (SOD1) leading to antiangiogenic and antitumour effects. The pharmacodynamics of tetrathiomolybdate has been followed by tracking ceruloplasmin (Cp), a biomarker for systemic copper. However, at least in mice, the inhibition of angiogenesis occurs before a measurable decrease in systemic copper is observed. Thus, the identification and characterisation of other biomarkers to follow the activity of ATN-224 in the clinic is of great interest. Here, we present the preclinical evaluation of two potential biomarkers for the activity of ATN-224: (i) SOD activity measurements in blood cells in mice and (ii) levels of endothelial progenitor cells (EPCs) in bonnet macaques treated with ATN-224. The superoxide dismutase activity in blood cells in mice is rapidly inhibited by ATN-224 treatment at doses at which angiogenesis is maximally inhibited. Furthermore, ATN-224 dosing in bonnet macaques causes a profound and reversible decrease in EPCs without significant toxicity. Thus, both SOD activity measurements and levels of EPCs may be useful biomarkers of the antiangiogenic activity of ATN-224 to be used in its clinical development.

Angiogenesis, the process of formation of new blood vessels from pre-existing ones, is critical for tumour growth and a promising therapeutic target for the treatment of cancer (Carmeliet, 2005; Doñate, 2005; Ferrara and Kerbel, 2005). Tetrathiomolybdate (TM) is an orally available copper-binding compound that has been shown to have efficacy as an antiangiogenic and antitumour agent in several mouse models of cancer (Pan *et al*, 2002, 2003a, 2003b; Lowndes and Harris, 2004; Goodman *et al*, 2005; Hassouneh *et al*, 2007), and has also been tested as an anticancer therapy in several clinical trials (Brewer *et al*, 2000; Redman *et al*, 2003). The ability of TM to inhibit angiogenesis has been attributed to the depletion of systemic copper, which has been described to affect multiple key regulators of angiogenesis (Brewer *et al*, 2000; Pan *et al*, 2002, 2003a, 2003b; Redman *et al*, 2003; Goodman *et al*, 2005; Hassouneh *et al*, 2007). ATN-224 is a second-generation choline salt of TM with improved stability, which has been tested in two phase I clinical trials (solid tumours and haematological malignancies) (Berenson *et al*, 2006; Lowndes *et al*, 2006) and is currently being investigated in three phase II trials (melanoma, prostate and multiple myeloma). We have recently identified the copper-dependent enzyme superoxide dismutase 1 (SOD1) as the main target for the antiproliferative activity of ATN-224 in endothelial and tumour cells (Juarez *et al*, 2006). Superoxide dismutase 1 is an abundant cytosolic enzyme that dismutates superoxide into hydrogen peroxide and molecular oxygen. The SOD1 activity in erythrocytes has been proposed as a biomarker for copper status in humans (Uauy *et al*, 1985; Milne, 1998).

Collectively, the data on TM and ATN-224 strongly support the notion that the biological activity of TM *in vivo* has a robust antiangiogenic component (Brewer *et al*, 2000; Pan *et al*, 2002, 2003a, 2003b; Redman *et al*, 2003; Lowndes and Harris, 2004; Goodman *et al*, 2005; Juarez *et al*, 2006; Hassouneh *et al*, 2007). Although ATN-224 has also been shown to have antiproliferative and/or proapoptotic effects on tumour cells *in vitro* (Juarez *et al*, 2006), it is yet unclear whether sufficiently high concentrations of ATN-224 can be achieved and maintained in the tumour environment *in vivo* to elicit those responses.

Ceruloplasmin (Cp) is a copper-containing oxidase, which is synthesised in the liver and circulated in blood. Cp contains approximately 95% of all copper in the blood; however, despite its high copper content, the role of Cp in copper transport is controversial. For example, targeted disruption of the Cp gene in mice does not alter copper absorption, transport or distribution (Meyer *et al*, 2001). Furthermore, patients with aceruloplasminaemia who lack a functional Cp have normal copper metabolism (Vassiliev *et al*, 2005). Although copper deficiency does not seem to directly affect the synthesis or secretion of Cp from the liver, the resulting apoprotein has a shorter half-life than that of the holo-Cp, and serum Cp measurements have been used to follow copper depletion: and pharmacokinetics (PK) and pharmacodynamics (PD) in clinical trials evaluating TM in cancer patients (Brewer *et al*, 2000; Redman *et al*, 2003; Lowndes and Harris, 2004; Goodman *et al*, 2005). However, the decrease in Cp levels may lag behind the onset of copper depletion, since apo-Cp is formed and degraded at a certain rate. Furthermore, we have observed that the inhibition of angiogenesis in mice by ATN-224 occurs before a measurable decrease in systemic copper is observed (Juarez *et al*, 2006). Thus, the identification of other biomarkers that may aid in the clinical development of ATN-224 is of interest.

## MATERIALS AND METHODS

### Reagents

ATN-224 (choline tetrathiomolybdate) was manufactured under cGMP using a proprietary manufacturing process with >99% purity. The ATN-224 stocks (50 mg ml^−1^) were prepared in water, and aliquoted and frozen until use. ATN-224 was diluted to the desired concentration using phosphate-buffered saline (PBS) or media just prior to use. Blood was drawn from human volunteers into Vacutainer tubes (BD Biosciences, San Jose, CA, USA) containing citrate. Plasma and blood pellets were obtained by centrifugation. Blood cells were washed twice with PBS and analysed for SOD activity. Bovine SOD1 was acquired from Sigma (St Louis, MO, USA).

### Matrigel plug

The Matrigel plug model was carried out as described before (Juarez *et al*, 2002). Briefly, cold Matrigel (BD) (500 *μ*l) was mixed with 800 ng ml^−1^ of FGF-2 or 300 ng ml^−1^ of VEGF and heparin (50 *μ*g ml^−1^). Negative control plugs did not contain the proangiogenic factors. The Matrigel mixture was injected subcutaneously (s.c.) into 4- to 8-week-old female BALB/c nude mice. Mice were treated by oral gavage either with distilled water or ATN-224. Animals were killed and the plugs recovered 5 days post plug injection. The haemoglobin levels in the plugs were determined using Drabkin's solution according to the manufacturers' instructions (Sigma).

### SOD assays

Blood pellets were lysed by adding an equal volume of RIPA buffer. A 1 : 20 dilution of the lysate was carried out in Tris-buffered saline (1 : 10 for plasma samples) and protein concentration determined by the Bradford assay (Bio-Rad, Hercules, CA, USA). Thirty micrograms (60 *μ*g for plasma samples) were assayed as follows: SOD1 activity was determined by measuring the inhibition of reduction of the water-soluble tetrazolium salt, WST-1 (2-(4-iodophenyl)-3-(4-nitrophenyl)-5-(2,4-disulfo-phenyl)-2*H*-tetrazolium, monosodium salt), which produces a water-soluble formazan dye upon reduction with a superoxide anion (Dojindo Molecular Technologies, Gaithersburg, MD, USA). Superoxide anion is generated by xanthine oxidase. Bovine SOD (Sigma), which has been shown to be equivalent to human SOD (Juarez *et al*, 2006), was used to generate a standard curve.

### Molybdenum levels

The concentration of ATN-224 in blood was determined by measuring molybdenum (Mo) using ICP-MS. Cell extracts or whole tissues were sent to ERI (Vancouver, BC, Canada) for analysis. Cells and tissues were digested using 6 N HNO_3_ to completely release all metals prior to analysis.

### Animal studies with mice

A431 cells from ATCC were grown in Dulbecco's modified eagle media, 10% fetal bovine serum at 37°C in a humidified 5% CO_2_ incubator. Two million cells were injected s.c. into female BALBc nude mice. When tumours reached 200–300 mm^3^, animals were randomised and treatment started. For some experiments, animals were treated daily with water, 50 or 150 mg kg^−1^ of ATN-224 by oral gavage. Animals were killed 10 days later, 3 h after the last dose of ATN-224. In other experiments, animals received water, or 100 mg kg^−1^ ATN-224 by oral gavage and were killed at different times. In both cases, tumours were isolated and blood was drawn. All animal procedures were performed according to approved protocols and in accordance with the recommendations for the proper care and use of laboratory animals. These studies were carried under the Institutional Animal Care and Use Committee (IACUC) from Perry Scientific (San Diego, CA, USA).

### Animal studies with bonnet macaques

#### Subjects

Subjects were three female and three male bonnet macaques (*Macaca radiata*), with a mean age of 12.9 years (s.e.=0.84), corresponding to early to mid-adulthood in this species. Subjects were singly housed in the same temperature- and humidity-controlled room. Lighting was on a 12 : 12 h light/dark cycle. Water and standard laboratory chow were available *ad libitum*.

#### ATN-224 administration

Cages had a movable back by which subjects could be brought to the front of the cage for injection of ATN-224 (0.5 mg kg^−1^, s.c.). Injections began with a different monkey each day. Monkeys initially received ATN-224 for 22 consecutive days, followed by a drug holiday for 26 days. Drug injections then resumed for 33 out of the next 35 days and ATN-224 was given 15 min before lights went off at night.

#### Blood sampling

Blood samples were always taken between 0900 and 1100 hours, and the order in which the subjects were sampled was systematically varied. The monkey was brought to the front of the cage by means of the movable cage back, and was given an injection of ketamine (10–15 mg kg^−1^, i.m.), immediately placed in a carrying cage, removed from the colony room and allowed to sit undisturbed for 10–15 min outside the veterinary treatment room a few feet away. The anaesthetised monkey was then taken from the carrying cage, the inner femoral area was shaved and disinfected, and approximately 10 ml of blood was withdrawn from the femoral triangle. The blood was deposited in tubes with and without EDTA, and placed on ice. If insufficient blood was drawn from the femoral triangle, additional blood was collected from the cephalic vein. All animal procedures were performed according to approved protocols and in accordance with the recommendations for the proper care and use of laboratory animals. These studies were approved by the IACUC from SUNY Downstate Medical Center.

#### EPC quantitation

Mononuclear cells were harvested from peripheral blood samples using Ficoll–Hypaque (Sigma) density-gradient centrifugation, followed by exposure of separated cells to a red blood cell lysis buffer (Roche Applied Science, Indianapolis, IN, USA). Direct immunofluorescence using combinations of monoclonal antibodies including anti-CD31-FITC (BD Biosciences); anti-CD133-PE (Miltenyi Biotec, Auburn, CA, USA) and anti-CD45-PE/Cy5 (eBioscience, San Diego, CA, USA) was performed as previously described (Zhang *et al*, 2005). Cells were analysed by three-colour flow cytometry using a FACSort flow cytometer (BD Biosciences) using the CellQuest software program (BD Immunocytometry Systems, San Diego, CA, USA) with appropriate compensation.

### Statistical analysis

GraphPad software was used for all statistical analysis. Data are presented as mean±s.d. Data were analysed using unpaired, two-tailed *t*-tests when comparing two variables. Analysis of variance with Tukey's posttest was used to compare data in experiments where more than two variables were compared simultaneously.

## RESULTS

**Figure 1:**
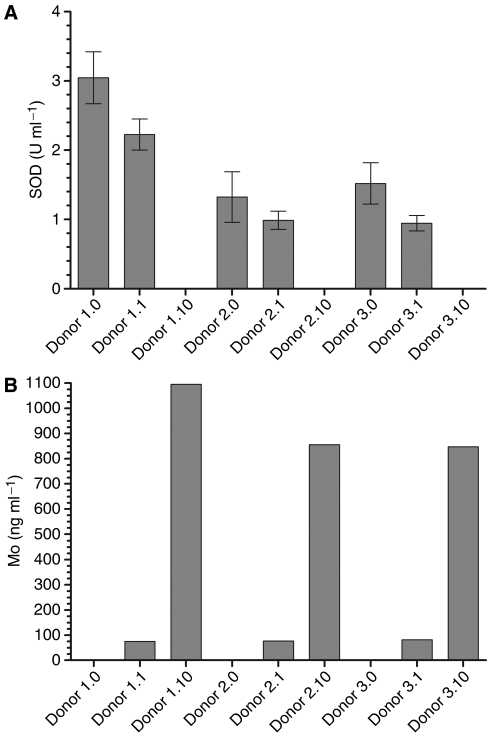
ATN-224 inhibits SOD activity in blood cells. Anticoagulated blood from three volunteers was incubated with ATN-224 (0, 1 and 10 *μ*M) for 5.5 h at 37°C, and plasma and blood pellets were prepared by centrifugation. (**A**) ATN-224 inhibits the SOD activity in blood pellets. The SOD activity was measured in blood pellets as described in Materials and Methods. (**B**) Levels of molybdenum are in agreement with SOD activity measurements. Molybdenum content was measured using ICP-MS for the same samples analysed for SOD activity.

**Figure 2:**
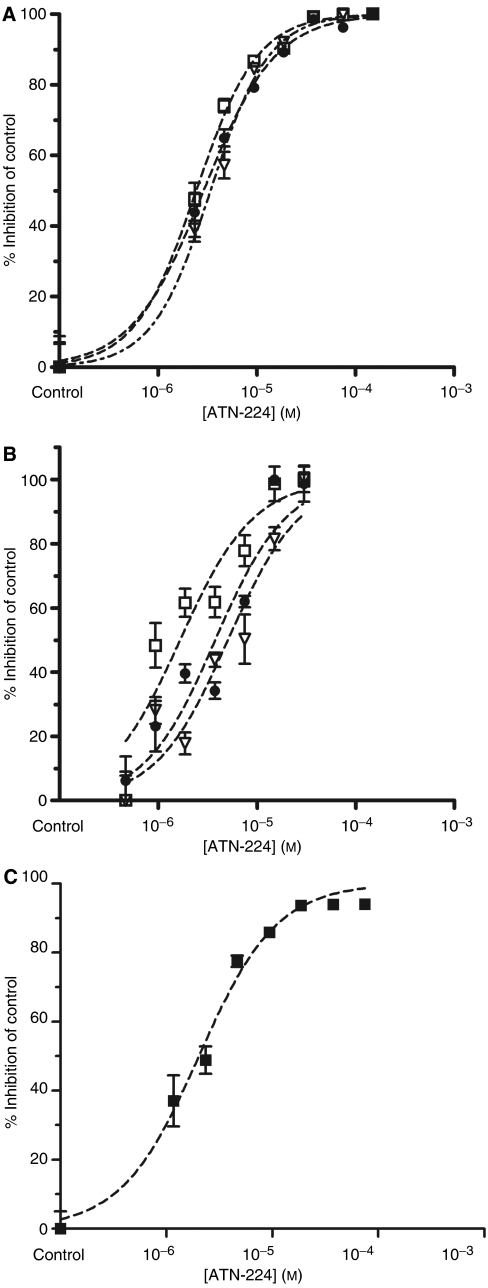
Inhibition of the SOD activity in human and mouse blood cells by ATN-224. (**A**) ATN-224 inhibits the SOD activity in human blood cells. Human anticoagulated blood from volunteers was incubated with different concentrations of ATN-224 overnight, and intracellular SOD activity was measured as described in Materials and Methods. Three different experiments are shown. ATN-224 inhibited SOD activity with an IC_50_=2.91±0.48 *μ*M. (**B**) ATN-224 inhibits the SOD activity of mouse blood cells. Mouse anticoagulated blood was incubated with different concentrations of ATN-224 overnight, and intracellular SOD activity was measured as described in Materials and Methods. Three different experiments are shown. ATN-224 inhibited SOD activity with an IC_50_=3.51±1.7 *μ*M. (**C**) The IC_50_ of ATN-224 for purified SOD1 activity increases in the presence of human plasma. Purified bovine SOD1 (5 U ml^−1^) was incubated with human plasma overnight, and SOD activity was measured as described in Materials and Methods. The SOD activity measured in human plasma is negligible. ATN-224 inhibited SOD activity with an IC_50_=2.03 *μ*M.

As ATN-224 treatment inhibits SOD activity in endothelial and tumour cells (Juarez *et al*, 2006), the effect of ATN-224 on SOD activity in blood cells was tested. Superoxide dismutase 3 is also a copper-dependent enzyme, which is bound to the extracellular matrix in mammalian tissues (Nozik-Grayck *et al*, 2005). It is mainly produced by vascular smooth muscle cells and retained in the vascular wall, and therefore mainly absent in erythrocytes and other blood cells (Nozik-Grayck *et al*, 2005). Superoxide dismutase 3 is mainly responsible for the SOD activity found in plasma and serum (Nozik-Grayck *et al*, 2005; Tasaki *et al*, 2006), and decreased copper levels inhibit its activity in rats (Johnson *et al*, 2005). Superoxide dismutase 3 activity in human plasma is low, but increases upon treatment with heparin (Tasaki *et al*, 2006). Blood from laboratory personnel who had volunteered for venipuncture was drawn and samples were incubated with ATN-224 for 5.5 h at 37°C. Plasma and blood pellets were prepared after incubation with ATN-224 and SOD activity and molybdenum (Mo) levels were determined. The levels of SOD activity in plasma from human volunteers were below the detection limits of our assay (data not shown) in agreement with the low levels of SOD3 that have been reported in plasma of humans (Tasaki *et al*, 2006). Figure 1A shows that ATN-224 inhibited SOD activity in blood cell pellets in a dose-dependent manner in agreement with the levels of Mo (representing ATN-224) associated with the cells (Figure 1B). ATN-224 inhibits SOD1 by removing copper from the active site (Juarez *et al*, 2006). As expected, it does not inhibit *Escherichia coli* SOD (data not shown), a manganese-containing enzyme. Furthermore, the majority of the SOD activity in blood cell lysates comes from SOD1 and not SOD2: as a SOD1-specific inhibitor (KCN) decreased the measured total SOD activity to background levels (data not shown). Thus, despite the fact that we used whole cell extracts, we only observed SOD1 in this assay. Next, the IC_50_ for the inhibition of SOD activity by ATN-224 in human and mouse blood cells was measured (Figures 2A and B) according to the protocol used in Figure 1, except that blood samples were incubated with ATN-224 overnight. The determined IC_50_ was approximately 3 *μ*M for human and mouse blood cell SOD1. The IC_50_ for inhibition of purified SOD1 is 300 nM (Juarez *et al*, 2006), which is ∼10-fold lower than the IC_50_ for SOD1 in blood cells. In the presence of albumin and presumably copper, ATN-224 forms a complex that is inactive in an endothelial proliferation assay (Juarez *et al*, 2006), and we suspected that the formation of that complex in blood could explain the higher IC_50_ against blood cells. This possible matrix effect in blood was tested by assaying purified bovine SOD1 in fresh human plasma instead of buffer and determining an IC_50_ for ATN-224 inhibition. The IC_50_ under these conditions was ∼2 *μ*M (Figure 2C), approximately seven-fold higher than that needed to inhibit purified SOD1, suggesting that a proportion of the ATN-224 in plasma is inactivated by forming a tripartite complex with albumin and copper. The data presented thus far supports the notion that ATN-224 inhibits SOD activity in blood cells and that the dose at which that occurs may reflect the level of active compound.

**Figure 3:**
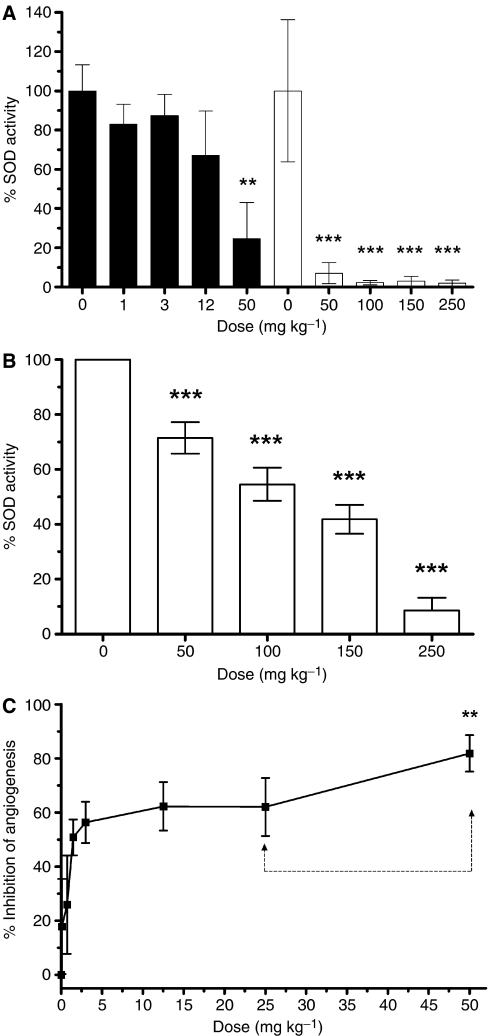
Plasma extracellular SOD, blood cell SOD and angiogenesis (Matrigel plug model) are inhibited in mice at different doses of ATN-224. (**A** and **B**) The ATN-224 dosing inhibits the SOD activity in plasma (**A**) and blood cells (**B**). Blood was collected after 5 days dosing with ATN-224, centrifuged and plasma and cell pellets were isolated. Superoxide dismutase activity was measured as described in Materials and Methods. In (**A**), two titrations (black and white bars) were carried out, and the 0 and 50 mg kg^−1^ data points are included in the second titration for comparison purposes with the first titration. (**C**) ATN-224 inhibits angiogenesis in the Matrigel plug model in a dose-dependent manner. Mice were treated daily for 5 days by oral gavage with the indicated amounts of ATN-224 or vehicle control. Angiogenesis was measured by measuring haemoglobin levels as indicated in the Materials and Methods section; ^**^P<0.01, ^***^P<0.001 (*t*-test).

Both in tumour models (Pan *et al*, 2002, 2003a, 2003b; Hassouneh *et al*, 2007) and in *in vivo* angiogenesis models, such as the Matrigel plug model (Juarez *et al*, 2006), ATN-224 has a strong antiangiogenic activity, and we wished to establish a relationship between the dose needed to inhibit angiogenesis and that needed to inhibit SOD activity in blood cells. Mice were dosed daily by gavage with different doses of ATN-224 for 5 days and SOD activity was determined in plasma and blood pellets at the end of the experiment (Figures 3A and B). In contrast to our results with human plasma (see above), mouse plasma SOD activity was easily detectable and ATN-224 treatment inhibited SOD activity indicating that SOD3, primarily responsible for SOD activity in plasma, can also be inhibited by ATN-224. In a second experiment, a dose titration for ATN-224 in the mouse Matrigel plug model was carried out (Figure 3C). The IC_50_ was determined to be approximately 1.50–3 mg kg^−1^ and a dose of 50 mg kg^−1^ produced an inhibition of 82% in the Matrigel plug assay. The 50 mg kg^−1^ dose is similar to a dose used before to optimally inhibit tumour growth and angiogenesis in tumour-bearing mice (35–50 mg kg^−1^ TM, which is equivalent to 59–85 mg kg^−1^ ATN-224 on a molar basis) (Pan *et al*, 2002, 2003a, 2003b; Hassouneh *et al*, 2007). Doses of 50 and 250 mg kg^−1^ were needed to completely inhibit SOD activity in plasma and blood pellets, respectively, suggesting that inhibition of SOD activity occurs at doses at which angiogenesis is maximally inhibited.

**Figure 4:**
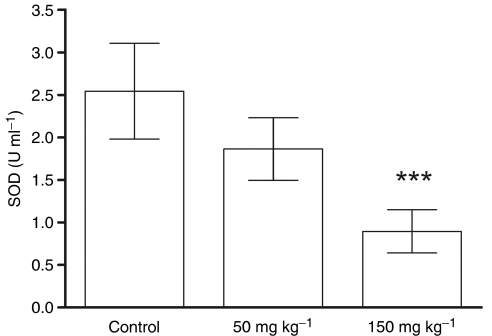
Inhibition of tumour cell SOD by ATN-224 in an A431 xenograft model. Tumour-bearing mice were treated daily for 10 days by oral gavage with the indicated amounts of ATN-224 or vehicle control. Tumours were minced and lysed. Superoxide dismutase 1 activity was measured as described in Materials and Methods; ^***^P<0.001 (*t*-test).

**Figure 5:**
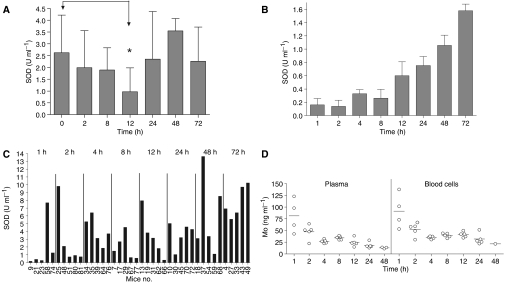
Inhibition of tumour cell SOD after a single dose of ATN-224 in A431 tumour-bearing mice. Mice were given a 100 mg kg^−1^ dose by oral gavage of ATN-224. At the indicated times, mice were killed and blood and tumours collected. (**A**) A single dose of ATN-224 inhibits the SOD activity in tumours in a time-dependent manner. Mice were killed at the indicated times and tumours resected, minced and lysed. Superoxide dismutase 1 activity was measured as described in Materials and Methods. (**B**) A single dose of ATN-224 inhibits the SOD activity in plasma in a time-dependent manner. (**C**) A single dose of ATN-224 inhibits the SOD activity in blood cells in a time-dependent manner; ^*^P<0.05 (*t*-test). (**D**) Levels of molybdenum agree with SOD activity measurements. Molybdenum content in plasma and blood cells was measured using ICP-MS.

The ATN-224 treatment also inhibited SOD activity in A431 tumours grown s.c. in mice (Figure 4). Unlike human tumours, experimental tumours grown s.c. in mice lack significant stroma, and therefore, the majority of the tumour mass is made up of tumour cells *per se*. Tumour-bearing mice were treated with buffer control, 50 or 150 mg kg^−1^ of ATN-224 daily by oral gavage for 10 days. The 50 mg kg^−1^ dose is similar to a dose used before to optimally inhibit tumour growth and angiogenesis in tumour-bearing mice (35–50 mg kg^−1^ TM, which is equivalent to 59–85 mg kg^−1^ ATN-224 on a molar basis) (Pan *et al*, 2002, 2003a, 2003b; Hassouneh *et al*, 2007). At the end of the treatment, the mice were killed, and the tumours were removed and lysed. The SOD activity in controls and ATN-224-treated mice was measured. The SOD assays were carried out using either 30 or 60 *μ*g of total extracted protein and the SOD activity (U ml^−1^) calculated from a standard curve generated using purified SOD as a standard. The 50 mg kg^−1^ dose of ATN-224 resulted in an approximately 26% reduction of the SOD activity, whereas the 150 mg kg^−1^ dose inhibited SOD by 65% (*P*<0.001) when compared with control mice. Similarly, when A549 tumour-bearing mice were treated for 10 days with 100 mg kg^−1^ daily of ATN-224, the SOD activity in the tumour was inhibited by ∼80% (data not shown). Next, we wished to investigate the kinetics of the SOD inactivation in tumours after a single dose of ATN-224 (100 mg kg^−1^) in A431 tumour-bearing mice. The results showed a significant inhibition of the SOD activity in the tumour, which was maximal at 12 h and returned to normal levels 24 h after treatment (Figure 5A). Similar results were observed in a second experiment (data not shown) in which the SOD activity and molybdenum levels in plasma and blood pellets were also measured (Figures 5B–D). As expected, the SOD activity in plasma is almost completely inhibited, but it appears to take longer to recover than in the tumour (Figure 5B). The SOD activity in the blood pellet is also inhibited (Figure 5C), but the level of inhibition is significantly variable among mice within the same time point. This variability is not due to differences in the level of drug as the concentration of Mo does not change significantly within mice in the same group (Figure 5D). This variability is not observed in mice that have been treated for 5 or 10 days with ATN-224. Altogether, these data indicate that SOD1 inhibition can be detected even after a single dose of ATN-224 in the tumour, plasma and blood cells, much earlier than the detection of decreases in Cp are observed in mice or humans (Lowndes and Harris, 2004; Goodman *et al*, 2005). This suggests that the SOD inhibition in blood and blood cells is a faster and more sensitive read-out of the biological activity of ATN-224. This is also consistent with our earlier observation that inhibition of angiogenesis takes place before systemic copper depletion is achieved (Juarez *et al*, 2006).

**Figure 6:**
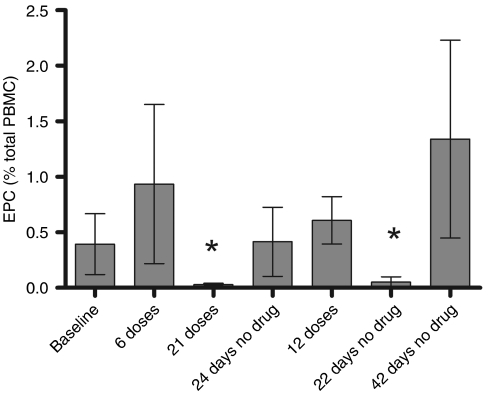
ATN-224 treatment lowers CECs and EPCs in monkeys. Six bonnet macaques were treated daily with ATN-224 (0.5 mg kg^−1^, s.c.) for 22 days followed by a 26-day drug holiday and 33 more days of treatment. The percentages of CD31+/CD133+/CD45 weak (EPCs) populations measured by flow cytometry are shown for each animal at the indicated time points on the *X* axis; ^*^P=0.021.

The levels of circulating endothelial cells (CECs) and endothelial progenitor cells (EPCs) increase in several pathological conditions, including cancer (Bertolini *et al*, 2006). Preclinical and clinical studies (Shaked *et al*, 2005; Zhang *et al*, 2005; Bertolini *et al*, 2006; Bhatt *et al*, 2007) have shown a correlation between CEC and EPC numbers and the antiangiogenic activity of different drugs, and thus, measurements of CEC and/or EPC have been proposed as biomarkers for monitoring antiangiogenic drug activity (Bertolini *et al*, 2006; Bhatt *et al*, 2007). To test the effect of ATN-224 on EPCs, different cell surface markers were first evaluated for the proper identification of EPCs using bonnet macaques as a model that would identify markers that could be translated into humans. Thus, EPCs were identified as CD31+/CD133+/CD45 weak and measured in six bonnet macaques that were treated daily with ATN-224 (0.5 mg kg^−1^, s.c.) for 22 days followed by a 26-day drug holiday, and then 33 more days of treatment. The dose was chosen based on bioavailability studies that showed bioequivalency of that dose with a dose of 25 mg kg^−1^ in mice given orally (data not shown). This dose was thought to be nontoxic, but sufficiently high to inhibit angiogenesis. The monkeys readily adapted to the experimental procedure, and exhibited no distress throughout. No behavioural changes (e.g., activity or interactional behaviours) were apparent across the course of the experiment, although mean body weight of the monkeys decreased by 8% by the end of the initial period of drug administration (*P*=0.012). By the end of the drug holiday, mean body weights had recovered to 95% of baseline values. It was thought that the drug could be interfering with the monkeys' evening feeding and during the second round of drug administration, ATN-224 was given 15 min before the colony room lights went off instead of the 30–90 min of the first round. Interestingly, mean body weights did not change during the course of the second round of drug administration. All animals developed mild alopecia during the first administration of ATN-224. This was partially resolved during the drug holiday and second round of ATN-224. The levels of EPCs increased initially at day 6 in five out of the six subjects, and then decreased to 7% of baseline at day 21 (Figure 6). The EPC levels rebounded to normal levels once treatment was discontinued and dropped again after treatment was reinstated. All other parameters measured, for example, red and white blood cell counts, platelets and haemoglobin levels were not affected by treatment (data not shown). Although we did not measure blood cell SOD in the macaque study, the recently completed phase I study with ATN-224 (Lowndes *et al*, 2006) indicated that substantial and maintained inhibition of blood cell SOD was associated with haematologic toxicities. As we observed significant depletion of EPCs in the macaques at a dose of ATN-224 that does not affect haematologic parameters, it is unlikely that we had significant inhibition of blood cell SOD in that study, further supporting the hypothesis that doses at which red blood cell (RBC) SOD activity is inhibited exceed the threshold required for antiangiogenic activity. Therefore, measuring EPCs in patients receiving ATN-224 may be a useful biomarker of antiangiogenic activity.

## DISCUSSION

The need for biomarkers in clinical development is clear and even more important for noncytotoxic agents, which often do not cause objective responses (Bhatt *et al*, 2007). Biomarkers are useful in managing dosing and toxicity, monitoring clinical benefit and/or in selecting patients more likely to benefit from the drug being tested. Ideally, besides being informative, biomarker assays should also be as minimally invasive as possible and preferably use tissue samples that are readily accessible in patients. Here, we report the evaluation of two possible biomarkers for ATN-224, the SOD activity in blood and EPC levels also in blood that will be measured during the clinical development of ATN-224. Both biomarker assays are minimally invasive, requiring only simple blood samples with minimal processing, and can be utilised to monitor biological activity of ATN-224 and to manage dosing. The SOD activity can be measured in blood pellets and tumour lysates, and is quickly inhibited upon ATN-224 dosing. The PK of ATN-224 is followed by tracking Mo in plasma (Berenson *et al*, 2006; Lowndes *et al*, 2006). This approach, however, does not distinguish the active compound from its metabolites. Despite our best efforts, no methodology has been found to track the active compound. An alternative approach could be to follow a pharmacodynamic marker in blood that may be a read-out of active drug exposure. The SOD activity in blood pellets of mice treated with ATN-224 reflects the levels of ‘active’ compound, and the changes are observed very early after the initiation of treatment in contrast to changes in Cp, which take weeks of TM treatment to be observed (Brewer *et al*, 2000; Pan *et al*, 2002, 2003a, 2003b; Redman *et al*, 2003; Goodman *et al*, 2005; Hassouneh *et al*, 2007). Thus, changes in the SOD activity in blood may provide a sensitive barometer of drug exposure in animals and humans.

The data in mice indicate that the concentrations needed to inhibit the SOD activity in blood pellets (ED_50_∼100–150 mg kg^−1^, equivalent to ∼8–12 mg kg^−1^ in humans) are higher than the doses that have been used in clinical trials (∼3 mg kg^−1^ of TM, equivalent to 5 mg kg^−1^ of ATN-224) (Brewer *et al*, 2000; Redman *et al*, 2003), implying that the SOD inhibition in human blood may not occur upon ATN-224 dosing. On the other hand, copper deficiency in humans has been shown to correlate with decreased SOD1 activity in RBC (Uauy *et al*, 1985; Milne, 1998), suggesting that ATN-224, which lowers levels of systemic copper in humans, should also inhibit SOD1. There are several possible reasons to explain this discrepancy between humans and mice. For instance, there may be differences among species as to the doses needed to inhibit the SOD1 activity in blood cells, and, importantly, we have shown that ATN-224 accumulates in cells (Juarez *et al*, 2006). This suggests that longer treatments than those used in this study may lower the threshold of ATN-224 needed for inhibiting blood cell SOD1. Indeed, data from a phase I trial showed a profound and persistent inhibition of SOD1 in blood pellets in patients treated at several ATN-224 doses below and at the maximal tolerated dose (Lowndes *et al*, 2006).

Inhibition of angiogenesis, as determined in the Matrigel plug model, occurs at lower doses than those needed for the SOD1 inhibition in blood pellets of mice. This suggests that the SOD1 inhibition in blood pellets will be indicative of antiangiogenic activity, since when a dose capable of inhibiting blood cell SOD1 is achieved the threshold for antiangiogenic activity would have been crossed. This is confirmed by the fact that at doses where SOD1 activity is beginning to be inhibited, there is already a significant effect observed on EPC levels, a biomarker for angiogenesis. In contrast, a lack of SOD1 inhibition would be noninformative with respect to the inhibition of angiogenesis. The relationship between the SOD1 inhibition in blood cells and antiangiogenic activity in humans has been studied in a phase I trial (Lowndes *et al*, 2006).

It has been shown that ATN-224 has antiproliferative and/or proapoptotic effects on tumour cells *in vitro* but only when SOD1 activity was inhibited almost 100% (Juarez *et al*, 2006). Inhibition of the SOD activity in tumour-bearing animals treated with ATN-224 for 10 days was 65–80% at a concentration that is approximately two- to three-fold higher than the concentrations reported for antitumour activity in mice, suggesting that ATN-224 may not be exerting a significant direct effect in tumour cells, at least in a xenograft model. Alternatively, it is possible that SOD1 inhibition is not homogeneous throughout the tumour and that areas exist in which SOD1 inhibition is complete and direct antitumour activity may occur. Currently, a method for measuring the SOD activity in fixed tissue is being developed to address this question. Finally, a xenografted tumour in a mouse, which is essentially a sphere of tumour cells, may not accurately reflect the situation with respect to human cancer, whether significant stromal components also exist in addition to the tumour cells themselves. Thus, our data only provide a rationale for studying the relationship of SOD1 inhibition to the inhibition of tumour growth in humans but may not accurately predict a correlation or lack thereof.

Endothelial progenitor cells have recently been used in several animal studies and clinical trials to follow the activity of a variety of antiangiogenic agents (Shaked *et al*, 2005; Zhang *et al*, 2005; Bertolini *et al*, 2006; Bhatt *et al*, 2007), and this technique continues to gain confirmation and acceptance as a promising biomarker for the inhibition of angiogenesis. On the basis of the results and rationale presented herein, SOD1 inhibition and depletion of EPCs/CECs, as well as Cp levels, have been evaluated for their ability to follow active drug and drug PD in a recently completed phase I clinical trial in patients with advanced solid cancer (Lowndes *et al*, 2006), and a paper describing these studies has now been submitted for publication.
